# The preferences of people in Australia to respond and engage with advertisements to promote reproductive health: Results of a discrete choice experiment

**DOI:** 10.1016/j.pmedr.2024.102657

**Published:** 2024-02-23

**Authors:** Edwina Dorney, Kirsten I Black, Marion Haas, Deborah Street, Jody Church

**Affiliations:** a Faculty of Medicine and Health, Central Clinical School, The Tavern, The University of Sydney, Medical Foundation Building K25, Sydney, New South Wales 2006, Australia; b Centre for Health Economics Research and Evaluation, Faculty of Health, University of Technology Sydney, New South Wales 2007, Australia

**Keywords:** Preconception, Reproductive health, Health promotion, Health communication, Discrete choice experiment, Consumer preferences

## Abstract

**Objectives:**

The health of people prior to pregnancy impacts pregnancy outcomes and childhood health, making the preconception period an important time to optimise health behaviours. Low awareness of the importance of this issue is a recognised barrier to achieving good preconception health. Public health messaging can help to address this barrier.

**Methods:**

A discrete choice experiment to assess the preferences of people of reproductive age for a health promotion advertisement for preconception health was conducted. Attributes of the advertisement image, title, additional text content and positioning, and the location of advertisement were assessed by fitting a mixed logit model to the choices made.

**Results:**

Three hundred and thirty-four responses were obtained, from people of reproductive age, both planning and not planning a pregnancy, in Australia. Participants placed most importance on the image, and the location in which they saw the advertisement. An image of adult and baby hands was preferred to adult hands only, and healthcare settings were preferred to more general media locations such as advertising online or on public transport. Preference was also given to the advertisement title of “Healthy you, Healthy baby”, closely followed by “Are you ready for pregnancy?”. The location and content of additional text did not significantly impact engagement with the advertisement.

**Conclusion:**

The image and title on the advertisement, and the locations in which they are placed were the most significant features to impact engagement with a health promotion advertisement for preconception health. This can inform health promotion efforts for preconception health.

## 1 Introduction

Health before pregnancy, or preconception health, impacts pregnancy outcomes, childhood health and the health of future generations. (Stephenson et al., 2018) Preconception care (PCC) aims to improve the health of women and their partners before pregnancy by identifying and addressing social, behavioural, and medical risk factors. (World Health Organization, 2013) A reported barrier to delivering PCC is a general lack of awareness of the importance of preconception health, (Bortolus et al., 2017) and a subsequent lack of presentation for preconception health checks. (Khan et al., 2019) Communicating the importance of preconception health with people who might want to have a child gives them the opportunity to improve their health before trying for a baby.

Improving preconception health is centred on the behaviour change of individuals, partners and communities, and the provision of information is a key requirement for change to occur. Heath promotion activities are an effective mechanism for information communication at a population level and can also have indirect benefits such as increasing social supports for behaviour change. (Wakefield et al., 2010) Australia has demonstrated previous success with population level health promotion campaigns in smoking cessation and skin cancer prevention fields. (Bayly et al., 2019, Walker et al., 2022) These campaigns were supported by additional interventions such as school and workplace education initiatives and fiscal measures recognising that education alone is not always sufficient to achieve behaviour change. Health promotion campaigns have the potential to increase individual and community awareness about the importance of preconception health and care.

Much of the previous evidence that focused on women shows that Australian women are keen to learn about preconception health and report a preference for online information sources. (Khan et al., 2019, Musgrave et al., 2023) As such, digital health solutions, including the use of online self-assessment tools are one potential enabler for the delivery of PCC. Self-assessment tools involve a self-completion activity on a given topic and then information and advice on the next steps to improve health. (Griffith University) The “Healthy you, healthy baby” tool, is a freely available online preconception health self-assessment tool housed on the YourFertilty website. Previously called The Healthy Conception Tool, this tool has been recently evaluated and enhanced with input from people of reproductive age across Australia to increase awareness of the importance of preconception health. This process was led by clinical, public health and policy researchers, and consumers with the input of a Rural Women’s Health Consumer Advisory Group (RWH-CAG), to ensure appropriate design to reach those who face geographical access challenges to care. (Black and Dorney, 2023) In- depth interviews and co-design activities as part of the enhancement process found that people of reproductive age want reproductive health digital tools that are easy to find, appealing and from credible sources. They also stated they were not familiar with the term “preconception care” and most would not have found the tool with the “Healthy Conception Tool” title. (Dorney et al., submitted for publication).

Developing an enhanced self-assessment tool is one step in the process of communicating the importance of preconception health. However, in the enhancement process, almost all interview participants stated that while they thought the tool was a good idea, that they would never have found it, and that it should be more widely and appropriately promoted. As such, health promotion, including the development of promotional material, to promote awareness and engagement with the enhanced tool, is also required. Currently, there is limited evidence that explores what influences a person of reproductive age to engage with a health promotion material on preparing for pregnancy.

The purpose of the current study was to gain an understanding into what features people value in a health promotion advertisement for a reproductive health self-assessment tool. A discrete choice experiment (DCE) will be used to assess the preferences of people of reproductive age for a health promotion advertisement for preconception health. In particular, what aspects of an advertisement would make them likely to engage with the self-assessment tool, thereby increasing knowledge and awareness about the importance of preconception health.

## 2 Methods

### 2.1 Study design

A DCE was conducted to understand the preferences of people of reproductive age in Australia for advertisements for a reproductive health self-assessment tool. Previous qualitative interviews highlighted the need to increase awareness of the self-assessment tool, with some individual suggestions for how this may be achieved. (Dorney et al., submitted for publication) A quantitative assessment, to understand the features of health promotion advertising and where this should be located, was required.

A DCE is a carefully constructed survey tool in which a hypothetical situation is described to respondents. Respondents are then shown sets of possible options, and for each set of options are asked to choose their preferred option given the hypothetical situation. The sets of options are known as choice tasks. Each option is described by attributes (features) and each attribute is presented at one of several possible levels. The choices made can be used to understand the preferences of respondents about the given attributes and their associated levels. This demonstrates how respondents are prepared to trade-off, or what they are willing to sacrifice in place for something else, across the presented attributes and levels. (de Bekker-Grob et al., 2012).

The attributes and levels used in a choice experiment can be decided upon by a review of existing literature and by scoping activities such as qualitative research to understand the attributes perceived to be relevant to a given health issue. (Reed Johnson et al., 2013) DCEs are traditionally used in health economics to understand the preferences of people for health interventions, inform health policy and program development. (Trapero-Bertran et al., 2019, Louviere and Lancsar, 2009) They have also been used to explore preferences for public health messaging. (Durvasula et al., 2019).

**Fig. 1:**
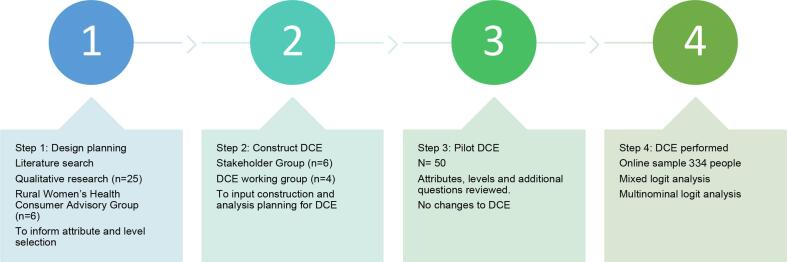
Stages of Discrete Choice Experiment (DCE) development for an advertisement about preconception health.

The design and development of the DCE involved several stages as outlined in Fig. 1.

### 2.2 Attribute and level development

The attributes and levels were informed by a review of current literature, consumer input from the RWH-CAG and qualitative research. This included in-depth interviews with 25 people of reproductive age on their perspectives of preconception health messaging. (Dorney et al., submitted for publication) These activities saw the identification of five key attributes to assess: image, name, text (that was seen as the “call to action” of the advertisement), positioning of text on the advertisement, and location where the advertisement is seen.

Selection of images as an attribute was informed from the qualitative interviews and then tested with the RWH-CAG. This process led to the decision to move away from images that depicted heteronormative relationships, defined age groups or implied socio-economic status. Two images, with hands only, where adult hands did not display wedding rings, were selected to be inclusive of all people of relationship statuses.

Qualitative interviews also showed that people were not familiar with the term “preconception care” with suggestions a plainer language term such as *“Are you ready for pregnancy?”* or “*Healthy baby”.* Interview participants also reported wanting more strengths based and empowering titles to encourage learning about preconception health. Possible titles were tested with the RWH-CAG, with four titles selected that tested across the words “*pregnancy”,* “*baby”*, and *“healthy”.*

Evidence demonstrates low levels of awareness of the importance of preconception health, particularly among those not planning a pregnancy. (Bortolus et al., 2017, Khan et al., 2019) Interview participants saw a key advantage, and drawcard of the self-assessment tool, being that it was quick to complete. These two messages informed the text on the advertisement to understand which message was more likely to invoke engagement with the advertisement.

In designing the advertisements, the research team identified the possibility for various locations of the text that was the call to action of the advertisement. Given the variability in this, this was added as an additional attribute to inform the development of the advertisement.

Evidence shows that people of reproductive age seek reproductive health information online, (Musgrave et al., 2023) and this was supported by the interview findings. The interviews also demonstrated appetite for self-assessment tools to be delivered in health care settings, such as primary care waiting rooms and as such this was included as a location for testing the advertisement.

### 2.3 Survey construction

The questionnaire was constructed in four sections. Section one collected background information about respondents. Section two consisted of an introductory task where participants first explored the preconception self-assessment tool, which was a mandatory requirement to complete the survey, and commented on additional desirable features for the Healthy Conception Tool. The choice tasks, with hypothetical situation, were completed in Section three, and Section four comprised a number of follow up questions that asked about the ease of completing the DCE, previous pregnancy information seeking, and name suggestions for the tool. The full DCE survey is available in Supplementary File A. Table 1 gives the attributes and levels for the DCE.

**Table 1:** Attributes and Levels for the Discrete Choice Experiment for an advertisement about preconception health.

Attribute		Level
1	Image	Adult hands in heart Adult’s and baby’s hands close
2	Names	Healthy you, healthy baby Be Baby Ready Are you baby ready? Are you ready for pregnancy?

3	Text	Learn about what you can do before pregnancy here In just a few minutes, learn how you can get ready for pregnancy here Even if you are not planning now learn what you can do for the future here Everyone needs to know. Learn how you can be ready for pregnancy here.

4	Location of name/text	Name top/text top Name top/text bottom Name middle/text bottom Name bottom/text bottom

5	Location of ad	Poster in GP waiting room Poster in Pharmacy Ad at bus stop Ad appears online Social media ad

### 2.4 Designed experiment

**Fig. 2:**
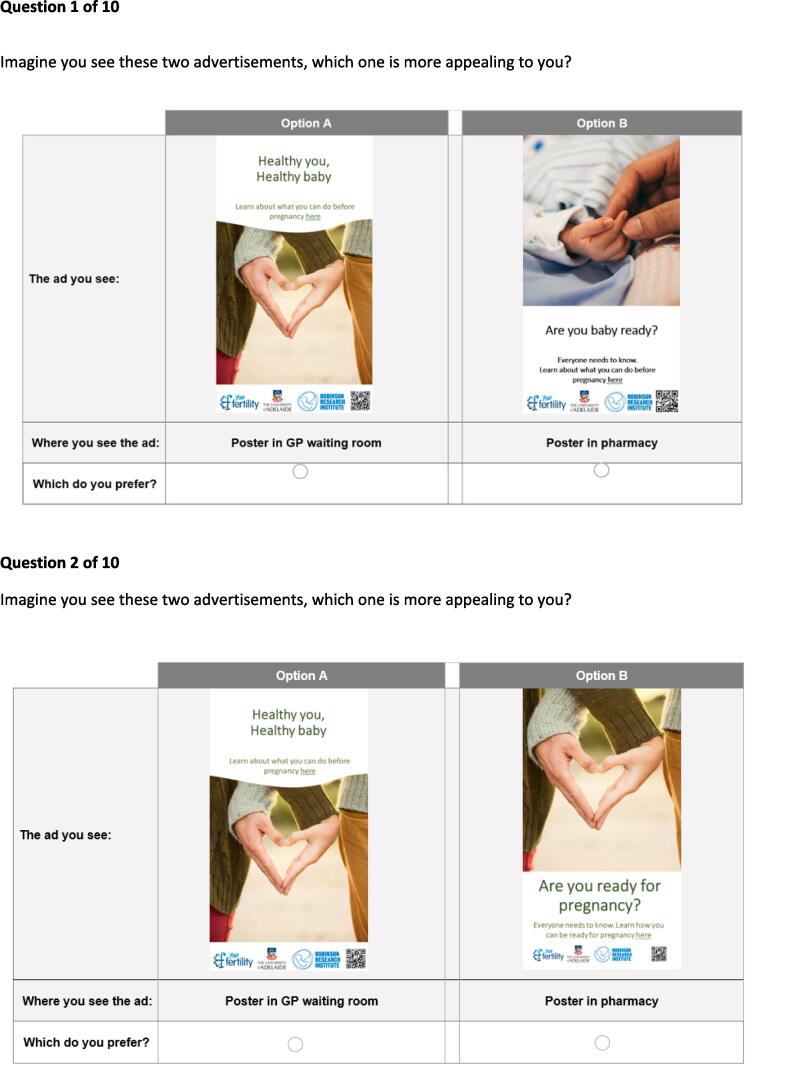
Choice Task examples for the Discrete Choice Experiment for an advertisement about preconception health.

Given the number of attributes and levels, 160 choice tasks were developed, divided into 16 versions of 10 choice sets each. The advertisement image, in which the first four attributes, image, name, text, location of name and text, were collapsed, resulted in a total of 116 distinct ads. The attribute of advertisement location was tested as an additional feature separate from the image. An example of a choice task is given in Fig. 2. Each respondent was randomly assigned to one of the 16 versions and completed 10 choice tasks, which were presented to respondents in random order. A pilot sample of 50 respondents was collected to check the data and test for survey consistency, and as this met requirements the survey proceeded to full launch without any changes.

The sample size was determined to allow for 20 responses per version. Although there are various approaches to determine sample sizes for DCEs, this has demonstrated reliable model estimates. (Lancsar and Louviere, 2008) Therefore, approximately 320 respondents were required.

### 2.5 Participants and setting

The DCE was administered by Pureprofile, an online panel provider with over 1 million panel members. Within Pureprofile, panel members complete surveys to earn points which can be exchanged for money or rewards. Panel members who were 18–41 years of age, living in Australia and able to speak and read English, were invited to participate via an email link which granted access to the survey. Respondents were recruited to be representative of the general population of Australia by age (within 18–41 years) and gender, and sample quotas were used to ensure recruitment of participants from rural and remote areas and from lower socio-economic backgrounds.

Panel members were asked indicate consent at the end of the introduction which contained information about the content and purpose of the study, and they could exit the survey and any point. Data were collected in January 2023.

### 2.6 Statistical Analysis

Descriptive statistics of the respondents were calculated, including socio-economic and demographic characteristics. All analyses were performed using R Statistical Software (v4.1.3; R Core Team 2021). (R Core Team, 2021) A multinomial logit (MNL) model was initially used to explore the preferences for the attributes and levels within the preconception health promotion advertisements. A mixed logit (MIXL) model was also used to explore preferences, as it allows for preference heterogeneity across respondents. (Train, 2009) The MIXL model was estimated using 2000 Halton draws and all attribute levels were considered to be random and independent parameters. Additional models were conducted using an interaction with gender to account for variations in responses between males and females. Likelihood-ratio tests were used to compare goodness of fit between different models.

### 2.7 Ethics

This study was approved by the University of Sydney Human Research Ethics Committee Project Number 2021/942 (18 July 2022) and meets the institutions guidelines for protections of human subjects in research.

## 3 Results

### 3.1 Respondent demographics

The final version of the survey was completed by 334 respondents; the participant demographics, and sexual and reproductive health demographics are shown in Table 2, Table 3 respectively. Respondents were representative of the Australian population with regards to gender and place of residence. Amongst respondents, 43 % (145/334) had children, 26 % (87/334) planned on having children in the next 12 months, and two-thirds reported having a regular general practitioner (GP).

**Table 2:** Participant Demographics Descriptive demographic statistics for people of reproductive age who participated in the Discrete Choice Experiment for an advertisement about preconception health in Australia 2023.

Characteristic	Number	Percentage	Australian Population Statistics %
Age (years)			
Range	18–41		
Mean	30.4		
Median	31		

Age Group			
18–24 years	84	25.2	26.1
25–29 years	50	15.0	21.0
30–34 years	99	29.6	22.3
35 – 41 years	101	30.2	30.4

Gender			
Female	167	50	49.7
Male	167	50	50.3

Place of residence			
Metropolitan	269	80.5	72.2
Regional	65	19.5	27.8

SEIFA ISRAD Quintile			*
Quintile 1 (most disadvantaged)	28	8.4	18
Quintile 2	54	16.2	19
Quintile 3	72	21.6	20
Quintile 4	83	24.8	21
Quintile 5 (most advantaged)	97	29.0	22

Indigenous Status			
Aboriginal or Torres Strait Islander	22	6.6	3.2
Neither Aboriginal of Torres Strait Islander	312	93.4	96.8

Highest level of education attained			
Year 11 or less	25	7.5	21.7
Year 12	55	16.5	17.9
Vocational	92	27.5	27.6
Tertiary	113	33.8	20.4
Postgraduate	49	14.7	12.3

*SEIFA ISRAD Quintiles reported for non-indigenous population.

**Table 3:** Sexual and Reproductive Health Demographics Descriptive sexual and reproductive health statistics for people of reproductive age who participated in the Discrete Choice Experiment for an advertisement about preconception health in Australia 2023.

Characteristic	Number	Percentage
Relationship Status		
Not currently in a relationship	94	28.1
Long term relationship	216	64.7
Casual relationship	24	7.2

Have Children		
Yes	145	43.4
Not currently in a relationship	189	56.6

Plan on having children in the next 12 months		
Yes	87	26.0
No	185	55.4
Don't know	62	18.6

Plan on having children in the future		
Yes	187	56.0
No	86	25.7
Don't know	61	18.3

Primary Healthcare – Regular GP		
Regular GP	224	67.1
Regular Practice but not same GP	80	24.0
Do not have Regular GP	30	9.0

Previously looked for information on getting healthy before pregnancy		
Yes	138	41.3
No	196	58.7

### 3.2 Analysis of choice tasks

Responses to the choice tasks were first analysed using a MNL model, and then a MIXL model. The MIXL model showed that there was significant heterogeneity in the preferences for the image and the location of where they saw the ad. The likelihood ratio test comparing the MNL model and MIXL model showed that the MIXL model provided an improvement in model fit (p-value of Chi^2^ test-statistic <0.001). (Table 4) Therefore this is the model that was used, the results of the MNL model are available in Supplementary File B.

**Table 4:** Results of the Mixed Logit (MIXL) Models Discrete choice experiment survey results, about an advertisement for preconception health, from people of reproductive age in Australia in 2023.

Attribute and Levels	Mean (SE)	Std. Dev (SE)
Image (Reference: adult hands in heart)		
Image: Adult and baby hands close	0.44 (0.06)	1.62 (0.14)***

Name (Reference: “Healthy you, Healthy baby”)		
Name: “Be Baby ready”	−0.27 (0.10)	0.55 (0.22) *
Name: “Are you baby ready”	−0.21 (0.10)	0.42 (0.27)
Name: “Are you ready for pregnancy?”	−0.13 (0.10)	0.52 (0.23)*

Text (Reference “Learn what you can do…”)		
Text: “In just a few minutes…”	−0.06 (0.10)	−0.02 (1.93)
Text: “Even if you are not…”	−0.03 (0.10)	−0.36 (0.34)
Text: “Everyone needs to know…”	−0.10 (0.10)	0.004 (1.87)

Text Location (Reference: Name top/Text top)		
Location: Name top/Text bottom	−0.17 (0.10)	−0.51 (0.26)*
Location: Name middle/Text bottom	−0.21 (0.10)	0.58 (0.25)*
Location: Name bottom/Text bottom	0.02 (0.10)	−0.01 (1.76)

Place (Reference: Poster in GP Waiting room)		
Place: Poster in pharmacy	−0.12 (0.10)	−0.28 (0.48)
Place: Ad at bus stop	−0.74 (0.12)	1.37 (0.21)***
Place: Ad appears online	−0.56 (0.11)	−0.58 (0.34)
Place: Social media ad	−0.53 (0.12)	1.21 (0.24)***
Log Likelihood		−2078.5
AIC		4212.93

Significance levels.

* p < 0.05.

** p < 0.01.

***p < 0.001.

AIC: Akaike Information Criterion.

**Fig. 3:**
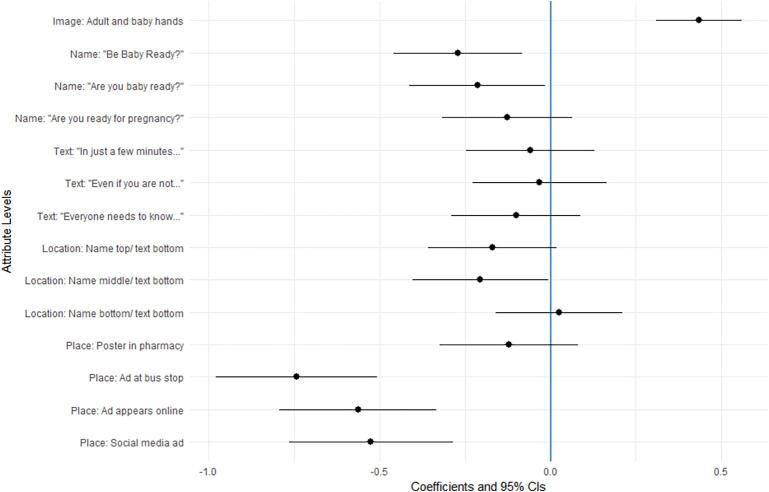
Preferences for features of preconception health advertisements.

The results of the MIXL model which accounted for heterogeneity in respondents’ preferences (Fig. 3) show that the image on the advertisement was the most important attribute and there was a strong preference for the image with adult and baby hands compared to adult hands only. The results for the location attribute indicate that respondents had strong preferences for the health care setting where they saw the advertisement. There was no significant difference in preferences between the advertisement being located in a GP surgery or a pharmacy. Showing the advertisement via social media, online advertising, or public advertising at a bus stop was significantly less popular than healthcare settings.

**Fig. 4:**
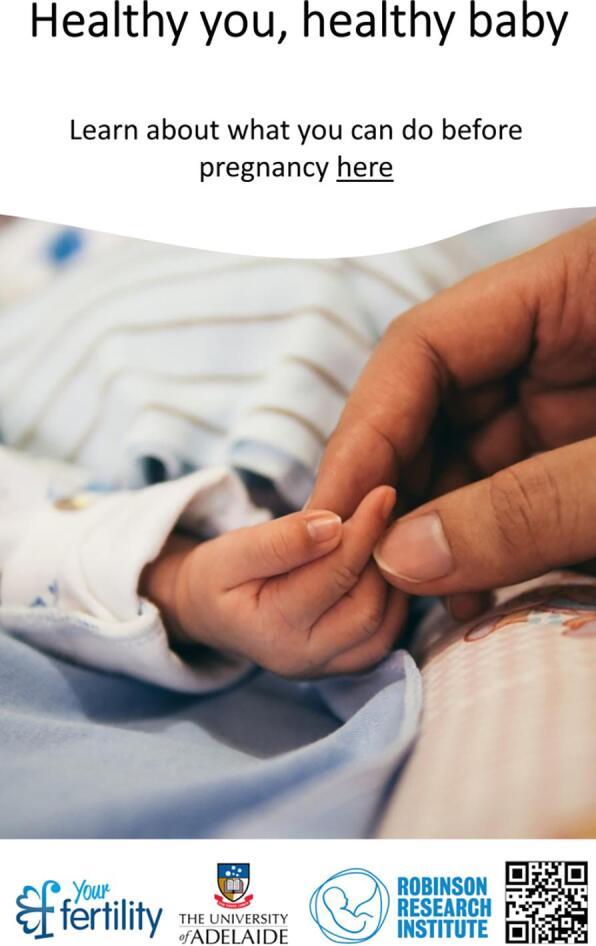
Preferred advertisement features.

The most popular name for the advertisement was *“Healthy you, healthy baby”* closely followed by *“Are you ready for pregnancy?”* with the other two names being significantly less popular. Regarding the location of the name and text within the advertisement, having the name and text together was slightly preferred to options with the name and text apart. Only the text attribute had no significant levels, indicating this attribute was not important to respondents. Additional models controlling for gender did not show any differences in preferences between males and females. Another model, controlling for people planning to have children in the future and those who are not planning was also performed. The only finding between these two groups was a slight difference in preferences for text placement. Fig. 4 demonstrates the preferred features of the advertisement as informed by the DCE.

### 3.3 Results of follow up questions

Results of follow up questions showed that people were keen to have additional opportunities to access preconception information, with 43 % (143/334) of respondents selecting additional services related to supporting links to free services and research trials. The next most popular features were options to sign up for email reminders and links to additional tools and directories for health care providers.

**Fig. 5:**
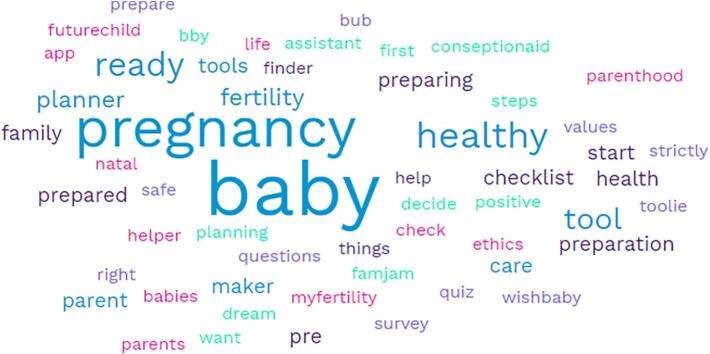
Word Cloud for suggested titles for preconception health tool.

Regarding the survey itself, most respondents (73 %, 244/334) indicated that they found the choice tasks either easy or extremely easy with only 4.2 % (14/334) finding the tasks difficult or extremely difficult. Open text comments about the survey also included positive feedback on the experience of completing the choice tasks. Many respondents (58.7 %, 196/334) indicated that they had not looked for information on getting healthy before pregnancy. Of those who had previously looked for information, most had looked online (85.6 %, 118/138) with GPs the next most frequent source (13.8 %, 19/138). Other sources were family, friends and government websites. Respondents were also invited to suggest names for the self-assessment tool, of the 55 suggested entries the most frequently cited words were “baby”, “pregnancy”, “healthy” (Fig. 5), which is consistent with the preferred titles from the DCE.

## 4 Discussion

This is one of the first studies to assess in detail the individual components of health promotion materials that are likely to influence engagement with educational materials for preconception health. A key step to enabling the delivery of PCC is health promotion to ignite individual and community awareness about the importance of preconception health. Currently, low levels of community awareness about preconception health, and a subsequent lack of presentation to health care providers for assessment and guidance are recognised barriers to PCC. (Khan et al., 2019).

Our study showed the choice of image significantly influenced the likelihood to engage with the preconception self-assessment tool. Use of images has been shown to increase attention to health information, and this is particularly important for people of low health literacy. (Houts et al., 2006) Increased impact on attention can be achieved if images are relevant to, and representative of, the target population. (Barros et al., 2014) As such it is recommended that consumer input is sought regarding the design of images for health communication to maximise reach and impact. (Sedeh et al., 2022).

The title on the advertisement material was also shown to significantly impact engagement, with respondents preferring a simple title of “Healthy you, healthy baby”. This was also supported in the free text responses of the survey. Preliminary research into designing the DCE survey found that the term “preconception care” was not recognised or understood by most people of reproductive age. Low health literacy levels have been shown to impact reproductive health knowledge, behaviours and outcomes. (Kilfoyle et al., 2016) This impact has been seen in relation to specific behaviours including accessing PCC and taking micronutrient supplements prior to and during pregnancy. (Endres et al., 2004, Poorman et al., 2014) These findings support the involvement of consumers in the design of health promotion material to ensure appropriate messaging that is easy to understand and resonates with the target audience.

The location for where respondents saw the advertisement was also a significant factor to drive engagement, with primary health care locations of General Practices or pharmacies preferred. This presents important opportunities to promote and offer PCC. Such opportunities have been piloted internationally where digital risk-assessment tools offered in primary care waiting rooms have been shown to increase the identification and assessment of preconception risk factors. (Montanaro et al., 2019).

While primary care locations were preferred, free text responses showed that most participants who had previously looked for preconception health information did so online. This is supported by existing evidence that shows young people use social media for reproductive health information. A recent Australian study showed that 32 % of women aged 18–24 use social media to access preconception health information, often at weekly intervals. (Skouteris and Savaglio, 2021) These patterns of use can inform targeted health promotion campaigns across both primary care locations and online sites.

The advertisement assessed in this study looks to target individuals and direct them to a self-assessment tool where they will explore their preconception health and enable behaviour change. Whilst this is important, health promotion campaigns should also include community and population health interventions to increase access and support for people to adopt positive preconception health behaviours. (Smith et al., 2017).

Increasing preconception health awareness in people not actively planning a pregnancy or “non-planners” is a significant challenge for preventive health. People planning a pregnancy are shown to be more receptive to preconception health information, where non-planners place greater significance on other priorities such as employment and financial stability. (Welshman et al., 2023).

It is increasingly recognised that involving consumers in health promotion design increases engagement and it has been recommended that such an approach underpins efforts in reproductive and maternity care. (Smith et al., 2017) This study has contributed to the enhancements to a previously existing online self-assessment tool for PCC. The name of the tool has been changed from “Healthy Conception Tool” to “Healthy you, Healthy Baby”, a title that is easier to understand and resonates with people of reproductive age. The findings will also inform health promotion activities to disseminate the tool in primary care.

Health promotion campaigns have been shown to be effective in changing health behaviour patterns, from smoking cessation to cancer screening, increasing physical activity and vaccination uptake. (Wakefield et al., 2010) With any behaviour change, complementary strategies are also needed to enable actions such as increased access to affordable healthy foods, green spaces and health care. PCC is no exception and collaboration across government and policy makers is also required.

### 4.1 Strengths and limitations

A strength of this study was a good balance of respondents who had previously looked for preconception health information and those who had not, and across those who were and were not planning on having children in the next 12 months. Given the significant rates of unplanned pregnancies in Australia and internationally, (Bearak et al., 2020) it is important to capture the preferences of non-planners, which has not been reported before. DCEs are limited by the number of attributes and levels that is practical to include in the survey design. As such, this study only tested two images. The testing of more images for different population groups is an area for future research. The study population was representative of the wider Australian population in all demographic attributes except socio-economic status and education level attained, which is often a feature of such surveys. Hence further investigation into health promotion requirements in people from lower socio-economic backgrounds is an area for future work. Areas for future research also include the preferences for people with a chronic condition as this is known to influence information seeking prior to pregnancy. (Hammarberg et al., 2022).

## 5 Conclusion

This study demonstrates what people of reproductive age value the most in a health promotion advertisement for preconception health. The advert image and title, and the locations in which the adverts are placed were the most significant features to impact engagement. This was tested across people both planning and not planning pregnancy in the next 12 months. These findings can inform health promotion efforts for preconception health, which is a key step in influencing behaviour change to improve reproductive health outcomes for all.

**Ethics approval statement**.

This study was approved the University of Sydney Human Research Ethics Committee Project Number 2021/942 (18 July 2022).

## CRediT authorship contribution statement

**Edwina Dorney:** Writing – review & editing, Writing – original draft, Software, Resources, Project administration, Methodology, Investigation, Funding acquisition, Formal analysis, Data curation, Conceptualization. **Kirsten I Black:** Writing – review & editing, Writing – original draft, Supervision, Project administration, Methodology, Investigation, Funding acquisition, Data curation, Conceptualization. **Marion Haas:** Writing – review & editing, Writing – original draft, Project administration, Methodology, Formal analysis, Data curation, Conceptualization. **Deborah Street:** Writing – review & editing, Writing – original draft, Project administration, Methodology, Formal analysis, Data curation, Conceptualization. **Jody Church:** Writing – review & editing, Writing – original draft, Software, Project administration, Methodology, Formal analysis, Data curation, Conceptualization.

## Declaration of competing interest

The authors declare that they have no known competing financial interests or personal relationships that could have appeared to influence the work reported in this paper.

## Data Availability

The data that has been used is confidential.
